# Enzymatically Crafted Bacterial Cellulose Nanoparticles Functionalized With Antimicrobial Peptides: Toward Sustainable Antimicrobial Formulations

**DOI:** 10.1002/biot.202400573

**Published:** 2025-02-24

**Authors:** Martina Schibeci, Rosa Gaglione, Noemi Russo, Raffaele Velotta, Bartolomeo Della Ventura, Angela Arciello

**Affiliations:** ^1^ Department of Chemical Sciences University of Naples Federico II Naples Italy; ^2^ Istituto Nazionale di Biostrutture e Biosistemi (INBB) Rome Italy; ^3^ Department of Physics “Ettore Pancini” University of Naples Federico II Naples Italy

**Keywords:** antimicrobial formulations, bacterial cellulose nanoparticles, cellulose enzymatic hydrolysis, host defence peptides, *Komagataeibacter xylinus*, sustainable production processes

## Abstract

Although natural antimicrobial peptides (AMPs) are endowed with excellent antimicrobial properties, only a few of them have been successfully translated to the market so far. This is mainly due to their short half‐life, to their high susceptibility to protease degradation, and to the lack of appropriate strategies for their efficient targeted delivery. Hence, the development of an effective system to deliver AMPs to the site of infection is urgent. The system here selected is represented by bacterial cellulose nanoparticles (BCNPs). Nanocellulose has recently emerged as one of the most promising “green” materials, attracting great attention due to its unique features, including biodegradability, sustainability, biocompatibility, and special physicochemical properties. To produce BCNPs, *Komagataeibacter xylinus* has been selected as host producing strain. Once obtained BC macrofibers, the production of BCNPs was set up by enzymatic hydrolysis using a commercial mixture of cellulases from *Trichoderma reesei* to develop a sustainable green biotechnological process. The storage stability of produced BCNPs has been also evaluated. Obtained BCNPs have been functionalized through non‐covalent bindings with an antimicrobial peptide previously identified in human apolipoprotein B and found to be endowed with strong antimicrobial properties in in vitro analyses and with good biocompatibility profiles when analyzed on human skin cells. This opens interesting perspectives to the applicability of the developed system in several biotechnological fields.

## Graphical Abstract and Lay Summary

Cellulose nanoparticles are gaining significant attention in biomedical and biotechnological applications. This study presents a sustainable and efficient method for producing Bacterial Cellulose Nanoparticles (BCNPs) through short‐time enzymatic reactions, eliminating the need for harsh substrate pretreatments or concentrated acids. The resulting BCNPs, characterized by their nanoscale size and length, show promising potential for drug delivery. Notably, these BCNPs were successfully loaded with natural Antimicrobial Peptides (AMPs) without requiring chemical modifications, offering a versatile and eco‐friendly drug delivery system for combating bacterial infections.

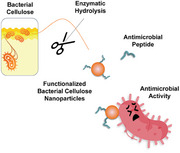

**Abbreviations**
AMPs: Antimicrobial Peptides
BC: Bacterial Cellulose (BC)
BCNPs: Bacterial Cellulose Nanoparticles
DLS: Dynamic Light Scattering
DMEM: Dulbecco's Modified Eagle's Medium
EDTA: Ethylenediaminetetraacetic Acid
ELS: Electrophoretic Light Scattering
FBS: Fetal Bovine Serum
HaCa: Human Keratinocytes
HDF: Human Dermal Fibroblasts
HS: Hestrin–Schramm Medium
IPTG: isopropyl‐b‐D‐thiogalactopyranoside
MHB: Mueller Hinton Broth
MTT: 3‐(4,5‐dimethylthiazol‐2‐yl)‐2,5 diphenyltetrazolium bromide
NB: Nutrient Broth
NC: Nanocellulose
QCM: Quartz Crystal Microbalance
TEM: Transmission Electron Microscopy
TSA: Tryptic Soy Agar
SEM: Scanning Electron Microscopy

## 1 Introduction

The global rise of infectious diseases is an increasingly significant health threat, projected to reach alarming proportions in the coming decades. The widespread misuse and overuse of antibiotics have contributed to the emergence and accumulation of drug‐resistant genes within bacterial populations [1]. This phenomenon has enabled numerous bacterial strains to develop resistance to many commonly used antibiotics. As a result, multidrug‐resistant pathogens are now responsible for life‐threatening infections, and their incidence is predicted to increase exponentially in the near future [2, 3, 4, 5].

A 2019 report from the World Health Organization (WHO) estimates that drug‐resistant bacterial infections currently cause over 700,000 deaths worldwide each year. If no decisive action is taken, this number is expected to rise dramatically to 10 million deaths annually by 2050 [6]. Given the severity of the situation, it has become imperative to develop innovative and effective antimicrobial strategies to combat this growing challenge.

Among the available alternative strategies, a promising avenue of research focuses on antimicrobial peptides (AMPs). These naturally occurring short polypeptides, typically composed of 12–50 amino acid residues, have demonstrated broad‐spectrum antimicrobial activity against bacteria, viruses, and fungi [7]. Due to their ability to penetrate bacterial cell membranes and target intracellular molecules, AMPs offer a dual mechanism of action for both antibacterial and antibiofilm applications [8, 9].

AMPs can be produced through both chemical synthesis and recombinant methods using microbial cells. The latter strategy typically involves fusing the specific AMP to a carrier protein or assembling AMPs into tandem arrangements to improve stability [10, 11, 12, 13, 14, 15]. Recombinant production of AMPs, particularly through prokaryotic cell factories, offers several advantages, including scalability, eco‐friendliness, and cost‐effectiveness, along with support for sustainable and circular economic practices [16, 17, 18, 19, 20]. Additionally, AMPs possess the general benefits of protein‐based therapeutics, such as excellent biocompatibility, biodegradability, and the ability to undergo functional and structural modifications via genetic engineering [21, 22].

However, despite their promising antimicrobial properties, the clinical application of AMPs has been hindered by their susceptibility to proteolytic degradation [23]. Consequently, developing effective systems to improve AMPs’ bioavailability, stability, and targeted action while ensuring sustained antimicrobial effects is crucial. To address these challenges, researchers have explored the use of nanocarrier systems for AMP delivery. By presenting AMPs as nanoscale entities, these systems offer enhanced tissue penetration, increased stability, and prolonged retention compared to their monomeric, soluble counterparts [24, 25, 26, 27]. Furthermore, the immobilization of AMPs on nanocarriers can enhance their biocompatibility towards mammalian cells, improve their in vitro stability, and maintain their promising antimicrobial activity [26].

Nanomedicine, which encompasses the development of nanoscale drug delivery systems, is rapidly becoming a key sector within the pharmaceutical industry. By 2030, the global nanomedicine market is expected to exceed $427 billion, driven by continuous investments in innovative technologies and the growing demand for safer, more effective therapeutic options [28].

Among the materials being explored for the development of nanocarrier systems, bacterial cellulose (BC) has emerged as a highly attractive option due to its abundance, renewability, and biocompatibility [28]. As the most abundant renewable natural polymer, BC has garnered increasing attention for its potential applications in nanomedicine [29, 30, 31, 32, 33, 34, 35, 36, 37, 38, 39]. Unlike plant‐derived cellulose, BC offers distinct advantages in terms of purity, mechanical properties, and ease of production [40]. Plant‐derived cellulose is often contaminated by hemicellulose, lignin, and other polymers, thus complex extraction and purification processes are required [41]. In contrast, BC is produced directly by bacteria, yielding a pure form of cellulose that does not require chemical treatments for its isolation [41].

The continuous synthesis of cellulose nanofibers by bacteria results in a unique three‐dimensional network structure stabilized by hydrogen bonds. This structure imparts BC with exceptional mechanical properties, including high crystallinity and an impressive Young's modulus, the highest among two‐dimensional organic materials [43]. Additionally, BC boasts a high specific surface area, which contributes to its high water retention and mechanical strength, making it a versatile material for biomedical applications. [44, 45]. BC's high tensile strength, flexibility, and permeability to gases and liquids further enhance its compatibility with living tissues, making it an ideal candidate for use in medical implants, wound dressings, and drug delivery systems [29, 30, 31, 32, 33, 34, 35, 36, 37, 38, 39].

Despite these advantages, large‐scale production of BC remains challenging due to the high costs of materials, particularly growth media, as wells as the significant energy requirements to maintain optimal bacterial growth conditions [46, 47, 48]. Nonetheless, efforts are underway to address these limitations by using low‐cost substrates derived from agro‐industrial and municipal waste, which could make BC production more economically feasible [49, 50, 51]. However, due to its high production costs, BC's widespread application is currently limited to high‐value markets.

Nanocellulose, a derivative of BC, has shown great potential as a drug delivery system due to its abundance of hydroxyl groups, which can bind active substances and water molecules, facilitating their controlled release at target sites [52]. The large specific surface area of nanocellulose also enables extensive surface modification, allowing for the functionalization of nanocellulose particles for a variety of biomedical applications. Covalent and non‐covalent modifications, such as oxidation, esterification, and polymerization, have been used to tailor the surface properties of nanocellulose for use in drug delivery systems [53, 54, 55]. In this study, a procedure was developed for the efficient production of bacterial cellulose nanoparticles (BCNPs) using the bacterial strain *Komagataeibacter xylinus* and Hestrin–Schramm (HS) medium. To develop a sustainable production process, enzymatic hydrolysis, rather than acidic hydrolysis, was employed to obtain BCNPs starting from BC fibers produced by bacterial cells. Commercial cellulases from *Trichoderma reesei* were used for this purpose. The resulting BCNPs were then functionalized through non‐covalent binding with a model antimicrobial peptide, r(P)ApoB_L_^Pro^, a recombinant peptide derived from human apolipoprotein B. The resulting BCNP‐AMP complexes were tested for their antimicrobial properties and found be highly effective on *S. typhimurium* ATCC14028, *B. globigii* TNO BM013, *S. epidermidis* ATCC35894, and *A. baumanii* ATCC19606 bacterial strains. Furthermore, the biocompatibility of BCNP‐AMP complexes was demonstrated through analyses on primary human dermal fibroblasts (HDFs) and HaCaT immortalized human keratinocytes.

Altogether, the results demonstrate that the developed system holds promising potential for future biomedical applications, particularly in the development of effective antimicrobial formulations.

## 2 Material and Methods

### 2.1 Materials

Glucose, peptone, yeast extract, disodium phosphate, citric acid, tryptone, sodium chloride, agar, sodium hydroxide (NaOH), sodium acetate, cellulases from *Trichoderma reesei* (aqueous solution ≥ 700 units/g), sulfuric acid, hydrogen peroxide, ampicillin, IPTG (isopropyl‐b‐D‐thiogalactopyranoside), ethylenediaminetetraacetic acid (EDTA), guanidine/HCl, β‐mercaptoethanol, hydrochloric acid (HCl), Trizma, acetic acid, Dulbecco's Modified Eagle's Medium (DMEM), fetal bovine serum (FBS), penicillin‐streptomycin (pen/strep), L‐glutamine, trypsin, 3‐(4,5‐dimethylthiazol‐2‐yl)‐2,5 diphenyltetrazolium bromide (MTT) and DMEM without phenol red were purchased from Merk Life Science S.r.l. (Milan, Italy). The Ni SepharoseTM 6 Fast Flow resin employed to perform the affinity chromatography was purchased from GE Healthcare Lifescience (Chicago, IL, USA). Mueller Hinton Broth (MHB) and Nutrient Broth (NB) were purchased from Becton‐Dickenson (Franklin Lakes, NJ, USA). The Quartz Crystal Microbalance (QCM) device employed was a Libra from Technobiochip (Italy). Bicinchoninic Acid (BCA) assay kit was purchased from Thermo Fisher Scientific (Waltham, MA, USA).

### 2.2 Bacterial Strains and Growth Conditions

The bacterial strain *Komagataeibacter xylinus* DSMZ 6513 was obtained from the German Collection of Microorganisms and Cell Cultures GmbH (Leibniz Institute DSMZ, Germany) and cultured in Hestrin‐Schramm (HS) medium (20 g/L glucose, 5 g/L peptone, 5 g/L yeast extract, 2.7 g/L disodium phosphate, 1.15 g/L citric acid). Bacterial strains *Acinetobacter baumannii* ATCC19606, *Salmonella typhimurium* ATCC14028, *Bacillus globigii* TNO BM013, and *Staphylococcus epidermidis* ATCC35894 were grown in Mueller Hinton Broth (MHB; Becton Dickinson Difco, Franklin Lakes, NJ, USA) and on Luria‐Bertani Agar (LBA; 10 g/L tryptone, 10 g/L yeast extract, 5 g sodium chloride, 15 g/L agar). In all the experiments, bacteria were inoculated and grown overnight in MHB at 37°C. The next day, bacteria were transferred to a fresh MHB tube and grown to mid‐logarithmic phase [11, 56, 57, 58, 59, 60, 61, 62].

### 2.3 Production of Bacterial Cellulose (BC) Macrofibers

BC production was performed by culturing *Komegateibacter xylinus* DSMZ 6513 in HS medium for 7 days at 30°C without shaking. Then, BC pellicle formed at the air‐liquid interface was harvested and treated with 0.1 M NaOH at 100°C for 30 min and subsequentially washed in Milli‐Q water. Cleaned BC pellicles were then pulped, freeze‐dried and ground to obtain BC macrofibers.

### 2.4 Bacterial Cellulose Nanoparticles (BCNPs) Production in Static Conditions

BC macrofibers (1 mg/mL) were hydrolyzed using 20 U/mL of a commercial mixture of cellulases from *Trichoderma reesei* (aqueous solution ≥ 700 units/g) in 0.1 M sodium acetate buffer at 40°C without shaking for various time intervals (3–6–9–24 h). At each selected time, an aliquot of the sample was withdrawn and diluted 1:10 (v/v) in Milli‐Q water prior to perform DLS analyses. BCNPs were collected by centrifugation at 4000 *g* for 30 min at 25°C. The obtained sediment was resuspended in Milli‐Q water and centrifuged again to perform a washing step. The washed BCNPs were then resuspended in the initial volume of Milli‐Q water and subjected to sonication in an ultrasound bath for 15 min. Samples were then diluted 1:10 (v/v) or 1:20 (v/v) to perform Dynamic Light Scattering (DLS) and Scanning Electron Microscopy (SEM) analyses, respectively.

### 2.5 BCNPs Production in Dynamic Conditions

As described above, BC macrofibers (1 mg/mL) were hydrolyzed by employing 20 U/mL of a commercial mixture of cellulases from *Trichoderma reesei* (aqueous solution ≥ 700 units/g) in 0.1 M sodium acetate buffer at 40°C under stirring at 200 rpm. At each selected time, an aliquot of the sample was withdrawn and diluted 1:10 (v/v) in Milli‐Q water to perform DLS analyses. At each selected time, BCNPs were collected by centrifugation at 10,000 *g* for 30 min at 25°C. The obtained sediment was resuspended in Milli‐Q water and centrifuged again to perform a washing step. The washed BCNPs were then resuspended in the initial volume of Milli‐Q water and subjected to sonication in an ultrasound bath for 15 min. Samples were then diluted 1:10 (v/v) or 1:20 (v/v) to perform DLS and electron microscopy analyses, respectively. To evaluate the effects of substrate concentration on the efficiency of enzymatic hydrolysis, increasing concentrations of BC macrofibers (1–2–4 mg/mL) were hydrolyzed for 24 h under stirring and collected as previously described by centrifugation at 10,000 and 14,000 *g* for 30 min at 25°C. In any case, washed BCNPs were subjected to sonication in an ultrasound bath for 15 min and diluted 1:10 (v/v) prior to performing DLS analyses.

### 2.6 Optimization of BCNPs Recovery

BCNPs were produced by hydrolyzing BC macrofibers (1 mg/mL) with 20 U/mL of cellulases from *Trichoderma reesei* in 0.1 M sodium acetate buffer at 40°C under stirring at 200 rpm. Obtained BCNPs were then recovered and washed by two steps of centrifugation at increasing *g* forces (4,000–8,000–10,000–14,000–27,000 *g*) for 30 min at 25°C. Washed BCNPs were resuspended in the initial volume of Milli‐Q water, subjected to sonication in an ultrasound bath for 15 min and diluted 1:10 (v/v) prior to performing DLS analyses. For all the experimental conditions under test, the amount of recovered BCNPs was evaluated by Quartz Crystal Microbalance (QCM) analyses.

### 2.7 Evaluation of BCNPs Storage Stability

To evaluate the stability of produced BCNPs, BC macrofibers (1 mg/mL) were hydrolyzed by using 20 U/mL of cellulases from *Trichoderma reesei* in 0.1 M sodium acetate buffer at 40°C under stirring at 200 rpm. Obtained BCNPs were harvested by centrifugation at 10,000 *g* for 30 min at 25°C. The obtained sediment was resuspended in Milli‐Q water and centrifuged again to perform a washing step. The washed BCNPs were then resuspended in the initial volume of Milli‐Q water and subjected to sonication in an ultrasound bath for 15 min. Obtained samples were then stored at 25°C for increasing time intervals (0–7–14–20–30 days). At each selected time, BCNPs were analyzed by DLS. Measurements were performed before and after a sonication of 15 min in an ultrasound bath. The storage stability of dried BCNPs was also evaluated by drying BCNPs collected by centrifugation either under a nitrogen flow or by lyophilization. In any case, the dried sediment was resuspended in the initial volume of Milli‐Q water and analyzed by DLS.

### 2.8 Analysis of BCNPs Dimension and Charge by Dynamic Light Scattering (DLS) and Electrophoretic Light Scattering (ELS)

The physicochemical properties of obtained BCNPs were monitored after appropriate dilution of the sample by DLS and ELS analyses performed at 25°C by using a Zetasizer Nano ZSP system (Malver, Worcestershire, UK). In any case, obtained results refer to two independent experiments, each one consisting of three repeated measurements.

### 2.9 Scanning and Transmission Electron Microscopy Analyses of BCNPs

To analyze BCNPs morphology, the samples were properly diluted and in any case an aliquot (15 µL) of the sample was spotted on a glass slide and air dried. The samples were then coated with a thin layer of Au‐Pd (Sputter Coater Denton Vacuum Desk V), then Scanning Electron Microscopy (SEM) analyses were performed by using a FEI Nova NanoSEM 450 (Fei Company, USA) at an accelerating voltage of 5 kV with Everhart Thornley Detector (ETD) and Trough Lens Detector (TLD) at high magnification. In the case of Transmission Electron Microscopy (TEM) analyses, an aliquot (4 µL) of a properly diluted sample was deposited on 200‐mesh carbon‐coated copper grids. Samples were then air‐dried and analyzed by using a FEI TECNAI G2 200 kV microscope (Fei Company, USA).

### 2.10 Evaluation of BCNPs Mass by Quartz Crystal Microbalance (QCM) Analyses

QCM analyses were performed to estimate the amount of produced and recovered BCNPs and to determine the percentage of the loading and the release of r(P)ApoB_L_^Pro^ antimicrobial peptide upon functionalization of produced BCNPs. The QCM device used is a Libra from Technobiochip, Italy. Employed AT‐CUT quartzes have a fundamental frequency of 10 MHz [63, 64] and the crystal and the gold electrode diameters are 1.37 and 0.68 cm, respectively [65]. As previously described [66], to perform the analyses, the gold surfaces were cleaned by immersing the oscillators in a Piranha solution (concentrated sulfuric acid and 40% hydrogen peroxide in a 5:1 v/v ratio) for 1 min. Afterward, the quartz crystals were washed with pure water. The entire cleaning procedure was performed in a fume hood. The gold‐quartz wafer was placed on the electronic console and the resonance frequency of the oscillator was monitored by using a proper software. Oscillation frequency changes (Δf) of the piezoelectric quartz crystal were related to the amount of mass adsorbed or desorbed from the sensor surface detected in real time and with high sensitivity [67]. All QCM measurements were performed at controlled temperature. To calibrate the QCM, known concentrations of peptide were dried on the electrode using serial dilutions, and the resulting frequency shift was recorded. Additionally, it was necessary to measure the control corresponding to the solvent alone, which was dried, and the value obtained was considered as the background to be subtracted from the measurements of the peptide‐containing samples. This allowed for the construction of a linear calibration curve and verification that the mass obtained, derived from converting the frequency shift to mass using Sauerbrey's equation [68], was consistent with the expected mass in the sample. The measurements were performed in duplicate.

### 2.11 Peptide Production

Expression and isolation of the recombinant peptide r(P)ApoB_L_^Pro^ was carried out as previously described [11]. Briefly, *E. coli* BL21(DE3) cells were transformed with a pET recombinant plasmid and grown in 50 mL of Luria‐Bertani (LB) medium containing 100 µg/mL of ampicillin, at 37°C up to an OD_600_ _nm_ of 2. These cultures were used to inoculate 1 L of LB/ampicillin medium. Cultures were incubated at 37°C up to OD_600_ _nm_ of 3.5–4. Expression of the recombinant protein was induced by the addition of IPTG (isopropyl‐β‐D‐thiogalactopyranoside) at a final concentration of 0.4 mM. Cells were harvested after overnight induction by centrifugation at 3,500 rpm for 30 min at 4°C and washed with 100 mM Tris‐HCl buffer pH 7.4. The bacterial pellet was suspended in 100 mM Tris‐HCl buffer pH 7.4 containing 20 mM ethylenediaminetetraacetic acid (EDTA) and sonicated in a cell disruptor (100% amplitude, 15 min, 30 s on, 30 s off, on ice). The suspension was then centrifuged at 12,000 rpm for 20 min at 4°C. The sediments containing the fusion protein were dissolved in 20 mL of denaturing buffer (5 M guanidine/HCl in 50 mM Tris‐HCl, pH 7.4) containing 10 mM β‐mercaptoethanol. The mixture was incubated at 37°C for 4 h under nitrogen atmosphere on a rotary shaker and then centrifuged at 12,000 rpm for 20 min at 4°C. Soluble fractions were then collected and purified by affinity chromatography on Ni Sepharose 6 Fast Flow resin (GE Healthcare Lifescience, Chicago, IL, USA). The chromatographic fractions were analyzed by 15% SDS‐PAGE, pooled, and extensively dialyzed against 0.1 M acetic acid pH 3.0 at 4°C. Any insoluble material was removed by centrifugation. The sample containing the fusion construct was then acidified to pH 2.0 by the addition of 0.6 M HCl to allow the cleavage of the Asp‐Pro linker peptide, purged with N_2_, and incubated at 60°C for 24 h in a water bath. The pH was then increased to 7.0–7.2 by the addition of 1 M NH_3_ and incubated overnight at 28°C to selectively precipitate the carrier ONC‐DCless‐H6, which is insoluble at neutral or alkaline pH values. The peptide was isolated from insoluble components by repeated cycles of centrifugation. A final gel‐filtration step was added to remove salts used along the purification process that tend to attach to the peptide molecules. Following this step, the peptide was lyophilized, and its purity was checked by SDS‐PAGE and mass spectrometry analyses. Lyophilized peptide was dissolved in pure water, unless differently specified, and quantified by BCA assay (Thermo Fisher Scientific, Waltham, MA, USA).

### 2.12 Preparation of BCNPs Loaded With r(P)ApoB_L_^Pro^ Antimicrobial Peptide

To load the produced BCNPs with the antimicrobial peptide under study, BCNPs were obtained in dynamic conditions and collected by centrifugation as described above. Washed BCNPs were then resuspended in a volume of Milli‐Q water five times lower than the initial volume of the sample, thus obtaining a concentrated solution of BCNPs (5× BCNPs). Sterilization of the sample was obtained by exposure to UV light for 15 min. Samples were then sonicated for 15 min in an ultrasound bath, to dissolve eventual BCNPs aggregates, and incubated with increasing concentrations of r(P)ApoB_L_^Pro^ peptide (0–100–200–300–350–500–1,000 µM) for 24 h under stirring at 100 rpm at 25°C. At the end of the incubation, the sample was centrifuged at 14,000 *g* for 30 min at 25°C, to separate BCNPs functionalized with peptide molecules (sediment) from the unbound peptide molecules (supernatant).

### 2.13 Evaluation of the Antimicrobial Activity of BCNPs Loaded With r(P)ApoB_L_^Pro^ Antimicrobial Peptide

BCNPs loaded with increasing concentrations of r(P)ApoB_L_^Pro^ antimicrobial peptide (0–100–200–300–350–500–1,000 µM) were collected by centrifugation. Afterward, the antimicrobial activity of the sediment (functionalized BCNPs) and of the supernatant (unbound peptide molecules) was evaluated by broth microdilution assay against *A. baumannii* ATCC19606 bacterial strain. To do this, the sediment (functionalized BCNPs) was resuspended in a volume of Milli‐Q water five times lower than the initial volume of the sample (5× BCNPs). Afterward, both the sediment and the supernatant were diluted 1:1 (v/v) into a solution containing 2 × 10^6^ CFU/mL of bacterial cells in 0.5× Nutrient Broth (NB; Difco, Becton Dickinson, Franklin Lakes, NJ, USA). Following an overnight incubation, each sample was diluted, plated on Luria‐Bertani Agar (LBA), and incubated at 37°C for 24 h, in order to count the number of colonies. BCNPs loaded with 350 µM r(P)ApoB_L_^Pro^ antimicrobial peptide were also tested on S. *thyphimurium* ATCC 14028, *B. globigii* TN0 BN013 and *S. epidermidis* ATCC 35894 bacterial strains by following the experimental procedure described above. Not loaded BCNPs were tested as controls.

### 2.14 Evaluation of Peptide Loading and Release by QCM Analyses

Produced 5× BCNPs were incubated with 350 µM r(P)ApoB_L_^Pro^ antimicrobial peptide for 24 h under stirring at 100 rpm at 25°C. At defined time intervals (0–1–2–4–8–24 h), aliquots of the sample were withdrawn, and collected by centrifugation at 14,000 *g* for 30 min at 25°C. In any case, the amount of unbound peptide in the supernatant was estimated by QCM analyses, to determine the percentage of peptide loading. At the end of 24 h incubation, the collected sediment containing the functionalized BCNPs was resuspended in the initial volume of Milli‐Q water and properly diluted to determine BCNPs charge by ELS and BCNPs morphology by SEM analyses. Loaded BCNPs were compared to unfunctionalized BCNPs tested as control. To evaluate peptide release, loaded BCNPs, once collected by centrifugation at 14,000 *g* for 30 min at 25°C after 24 h of incubation with 350 µM r(P)ApoB_L_^Pro^ antimicrobial peptide, were resuspended in the initial volume of Milli‐Q water and incubated for 24 h under stirring at 100 rpm at 25°C. At defined time intervals (0–1–2–4–8–24 h), aliquots of the sample were withdrawn, and collected by centrifugation at 14,000 *g* for 30 min at 25°C. In any case, the amount of released peptide molecules in the supernatant was estimated by QCM analyses.

### 2.15 Analysis of the Biocompatibility of BCNPs Loaded With r(P)ApoB_L_^Pro^ Antimicrobial Peptide

To analyze the biocompatibility of BCNPs loaded with r(P)ApoB_L_^Pro^ antimicrobial peptide, effects on the viability of immortalized human keratinocytes (HaCaT) and of primary human dermal fibroblasts (HDF) were evaluated. To this purpose, cells were cultured in Dulbecco's Modified Eagle's Medium (DMEM) supplemented with 10% fetal bovine serum (FBS), 1% antibiotics (pen/strep), and 1% L‐glutamine at 37°C in a humidified atmosphere containing 5% CO_2_. Cells, once confluent, were detached by using trypsin and 500 µL of cell suspension were seeded into a 96‐well microtiter plate (5 × 10^3^ cells/well). Following an incubation overnight at 37°C, either 5× BCNPs loaded with 350 µM antimicrobial peptide and collected by centrifugation (sediment) or the unbound peptide molecules (supernatant) were added on the cells and the samples were incubated at 37°C for 24 h. Unloaded BCNPs and the not centrifuged sample were also tested as controls. Cytotoxicity was then evaluated by a 3‐(4,5‐dimethylthiazol‐2‐yl)‐2,5 diphenyltetrazolium bromide (MTT) reduction inhibition assay. To do this, following 24 h of incubation with the samples under study, the medium was removed and 300 µL of MTT reagent (0.5 mg/mL), dissolved in DMEM without phenol red, were added to the cells. The procedure was then carried out as previously described [69]. Cytotoxicity experiments were performed at least three times independently. Cell survival was expressed as the percentage of viable cells in contact with samples under study with respect to untreated cells.

### 2.16 Statistical Analyses

Statistical analyses were performed using Student's *t*‐test. Significant differences were indicated as: * (*p* < 0.05), ** (*p* < 0.01), *** (*p* < 0.001), or **** (*p* < 0.0001)

## 3 Results and Discussion

### 3.1 Optimization of the Production and Recovery of Bacterial Cellulose Nanoparticles (BCNPs)

BC was obtained by culturing *Komegateibacter xylinus* DSMZ 6513 cells in liquid HS medium (20 g/L glucose, 5 g/L peptone, 5 g/L yeast extract, 2.7 g/L disodium phosphate, 1.15 g/L citric acid) for 7 days at 30°C without shaking. Once a pellicle formed at the air‐liquid interface, BC was cleaned, pulped, dried, and ground to obtain BC macrofibers (Figure S1). To produce BCNPs, enzymatic hydrolysis was chosen as a sustainable alternative to traditional acid hydrolysis method. For this purpose, a commercial cellulase mixture from *Trichoderma reesei* was used.

#### 3.1.1 Optimization of the Production of BCNPs in Static Conditions and Their Recovery

**FIGURE 1:**
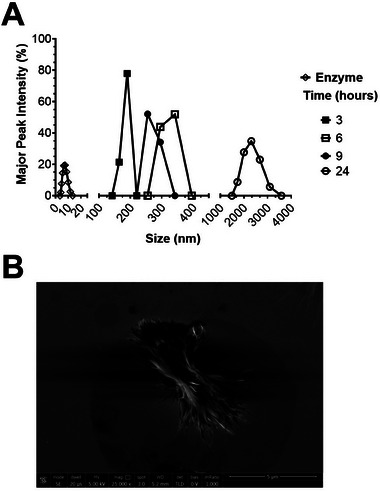
Production of BCNPs via enzymatic hydrolysis in static conditions. (A) The major peak intensity (%) revealed by DLS analyses of enzyme and BC macro‐fibers subjected to enzymatic hydrolysis as a function of the size. (B) SEM analysis of BCNPs obtained after 24 h of hydrolysis (scale bar 5 µm).

BCNPs production and recovery were performed under static conditions (in the absence of shaking). The kinetics of hydrolysis were investigated using DLS analyses, evaluating the following parameters: major peak intensity (%), major peak size (nm), Z‐average size (nm), defined as the harmonic intensity average particle diameter, and the PolyDispersity Index (PDI), which serves as an indicator of sample's quality in relation to size distribution (Table 1). As a control, a sample containing the enzyme without the substrate was also analyzed by DLS to exclude the presence of peaks related to the enzyme alone. The analyses revealed a single peak for the enzyme alone, with a mean dimension of 8 ± 2 nm (Figure 1A).

**TABLE 1:** Kinetic analysis of BCNPs production in static conditions by Dynamic Light Scattering.

Time (hours)	Major peak intensity (%)	Major peak size (nm)	Z‐average (nm)	PDI
3	99 ± 1	184 ± 8	1161 ± 61	0.974 ± 0.037
6	96 ± 6	320 ± 8	1018 ± 42	0.845 ± 0.112
9	86 ± 19	271 ± 2	925 ± 61	0.785 ± 0.084
24	100	2463 ± 237	3223 ± 259	0.363 ± 0.019

Time‐dependent analyses of the sample subjected to hydrolysis under static conditions revealed that both the major peak and overall size increased over time, with the homogeneity of the sample also improving (Table 1). Specifically, the Z‐average value was found to be 1161 ± 60.8 nm after 3 h of hydrolysis and 3223 ± 259 nm after 24 h of hydrolysis (Table 1). This trend in the Z‐average was accompanied by a corresponding decrease in the PDI, which was 0.974 ± 0.037 after 3 h and 0.363 ± 0.019 after 24 h (Table 1). A schematic of the obtained results is shown in Figure 1A, where large clusters of particles with similar sizes can be observed after 24 h of hydrolysis. Given that the sample appeared homogeneous in size after 24 h of hydrolysis, this experimental condition was selected for further analyses.

SEM analyses were conducted and revealed the presence of undigested BC macrofibers (approximately 5 µm in length) alongside BCNPs of various sizes (Figure 1B), consistent with the DLS data. Overall, the data indicated the need to optimize the hydrolysis conditions. Once the hydrolysis was complete, a centrifugation step was preferred over heat treatment to stop the reaction and separate BCNPs from cellulases, as it allows for the recovery of the enzyme in its active form. This approach opens up promising possibilities for developing a sustainable process with enzyme recycling, significantly reducing production costs.

BCNPs were collected by centrifugation (4,000 *g* for 30 min), and the resulting supernatant (reaction supernatant) was retrieved. The sediment containing BCNPs was resuspended in an equal volume of Milli‐Q water and centrifuged again under the same conditions (4,000 *g* for 30 min) to remove any remaining traces of the enzyme in the supernatant (wash supernatant). The sediment was then diluted 1:10 (v/v) in Milli‐Q water and subjected to sonication in an ultrasonic bath for 15 min to dissolve large BCNPs aggregates.

DLS analyses of the reaction supernatant revealed objects with dimensions comparable to those of the enzyme molecules (8 ± 1 nm) and a second peak with lower intensity, characterized by mean dimensions of 580 ± 278 nm (Table S1). Analysis of the wash supernatant confirmed the effective separation of BCNPs from enzyme molecules (Table S1), though a small amount of BCNPs (mean size of 410 ± 327 nm) was still present in the wash supernatant (Table S1). As shown in Table S1, analysis of the obtained sediments confirmed the presence of BCNPs with a size of 767 ± 27 nm, with no peak corresponding to the enzyme observed in the sediment (Table S1).

These findings suggest that the proposed centrifugation method is effective for fully separating the active form of the enzyme from the BCNPs. Future experiments will explore the possibility of using the recovered enzyme for serial reactions, which could lead to a more sustainable method for BCNPs production.

#### 3.1.2 Optimization of the Production of BCNPs in Dynamic Conditions and Their Recovery

**FIGURE 2:**
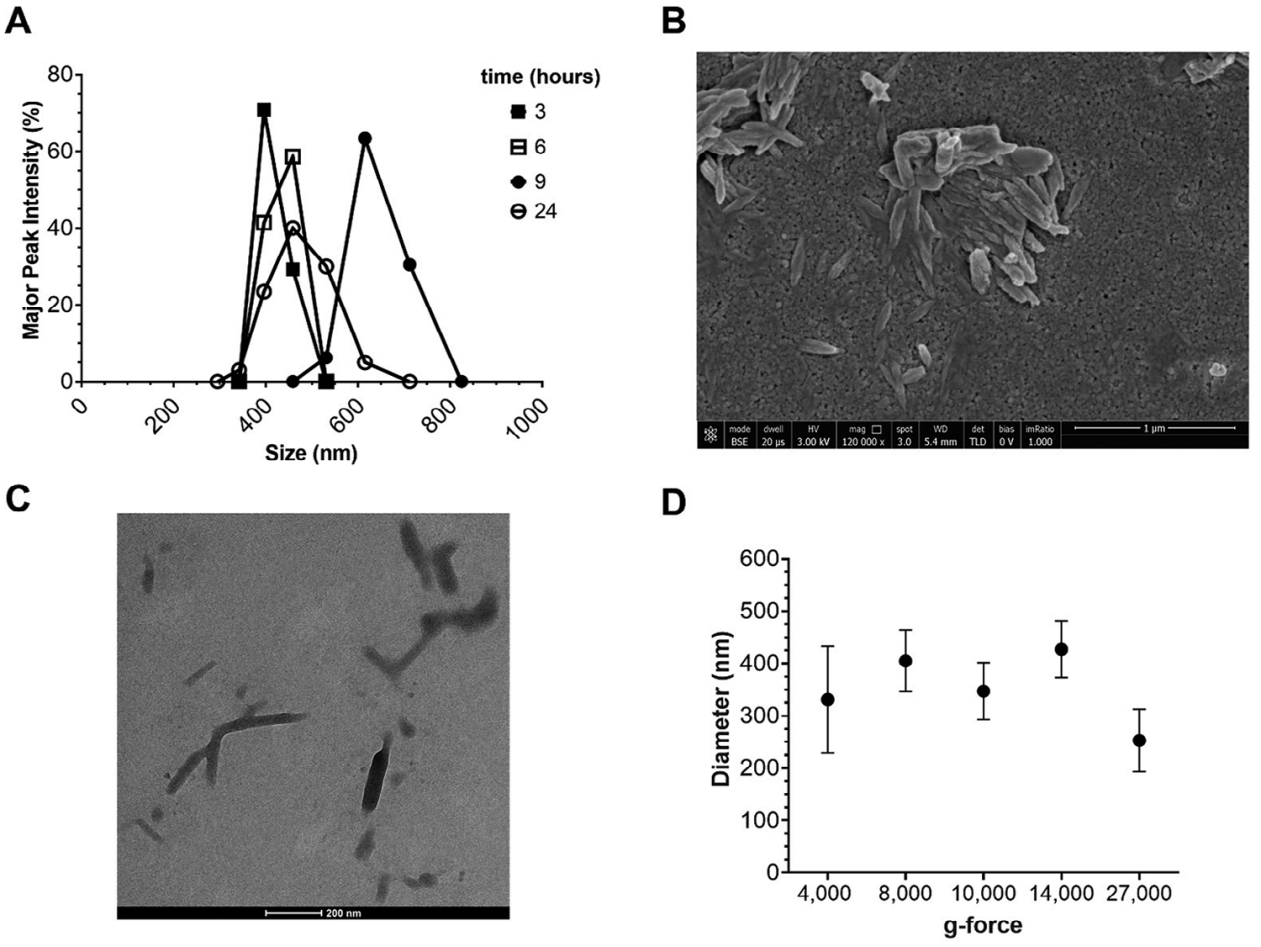
Production of BCNPs via enzymatic hydrolysis in dynamic conditions. (A) The major peak intensity (%) revealed by DLS analyses of BC macro‐fibers subjected to enzymatic hydrolysis as a function of the size of obtained BCNPs. (B) SEM analysis of BCNPs obtained after 24 h of hydrolysis in dynamic conditions (scale bar 1 µm). (C) TEM analysis of BCNPs obtained after 24 h of hydrolysis in dynamic conditions (scale bar 200 nm). (D) Dimension of BCNPs at different g‐forces upon enzymatic hydrolysis in dynamic conditions; in any case, the mean dimension (nm) of collected BCNPs is reported as a function of applied *g*‐force.

The experimental procedure described above was also used to produce BCNPs via enzymatic hydrolysis under stirring at 200 rpm (dynamic conditions). Time‐course analyses were carried out using DLS after 3–6–9–24 h of incubation. As shown in Table 2, the major peaks sizes were found to be 380 ± 63, 404 ± 106, and 682 ± 235 nm after 3, 6, and 9 h of incubation, respectively. These values are larger than those obtained under static conditions (Table 1). After 24 h of incubation under dynamic conditions, the major peak showed a size of about 477 ± 199 nm, with a Z‐average of 3256 ± 1716 nm (Table 2 and Figure 2A), suggesting the presence of large aggregates, as expected from previous results.

**TABLE 2:** Kinetic analysis of BCNPs production in dynamic conditions by Dynamic Light Scattering.

Time (hours)	Major peak intensity (%)	Major peak size (nm)	Z‐average (nm)	PDI
3	100	380 ± 63	1458 ± 100	0.881 ± 0.097
6	100	404 ± 106	1225 ± 834	0.714 ± 0.403
9	98 ± 4	682 ± 235	1938 ± 703	0.643 ± 0.428
24	84 ± 16	477 ± 199	3256 ± 1716	0.949 ± 0.129

The comparison between the enzymatic hydrolysis reactions performed under static and dynamic conditions indicates that stirring reduces the tendency of the BCNPs to aggregate. To recover the produced BCNPs, the hydrolysis reaction mixture was subjected to centrifugation (10,000 *g* for 30 min) and sonication (15 min). In contrast to the sediments obtained under static conditions, the BCNPs in the sediment from centrifugating the reaction mixture under dynamic conditions showed significantly smaller sizes (Table S2), with particles ranging from about 250–400 nm at both investigated time points. The Z‐average values varied considerably over time, shifting from 2282 ± 1683 nm at 3 h to 1607 ± 301 nm at 24 h of hydrolysis under dynamic conditions, respectively (Table S2). This change might be attributed to partial digestion of the sample after 3 h of hydrolysis, leading to higher variability in particle sizes, as shown in Figure S2, where cellulose fibers are still present after 3 h of hydrolysis under dynamic conditions. Despite the smaller size of BCNPs after 3 h of hydrolysis under dynamic conditions, unhydrolyzed microfibers seem to be still present (Figure S2).

SEM was also performed on the sample subjected to full hydrolysis (24 h of hydrolysis under dynamic conditions) followed by centrifugation at 10,000 *g* for 30 min to efficiently collect the BCNPs (Figure 2B). Aggregates of BCNPs were visible in the SEM image (Figure 2B). This aggregation may result from both the intrinsic tendency of BCNPs to aggregate and the methodology used for preparing the sample for SEM analysis. The procedure requires drying the sample on glass coverslips until complete solvent evaporation, which could promote aggregation. However, it is important to note that BCNPs’ tendency to aggregate was also observed in TEM analysis (Figure 2C). Similarly, the aggregation may be due to the inherent properties of the BCNPs or to the sample preparation method, which also involves drying until complete solvent evaporation.

It is important to mention that DLS, used to compare the effectiveness of the enzymatic hydrolysis reaction under different experimental conditions, may not be the most reliable technique for obtaining precise measurements of rod‐shaped nanoparticles. Nonetheless, DLS is widely accepted for comparative analysis of non‐spherical nanoparticle dimensions. To complement the DLS findings, we also performed SEM and TEM observations.

Based on the results, it must be emphasized that the observed increase in particle size, especially for the hydrolysis reaction performed under dynamic conditions, primarily reflects variability in the Z‐average values obtained after 24 h of hydrolysis. The recorded values represent the mean size of the entire particle population detected in the samples, irrespective of their relative abundance. Since BCNPs are known to have a high propensity to aggregate, it is likely that the increase in the number of nanosized BC particles led to a higher aggregation rate, which may have contributed to the shift in Z‐average values toward larger sizes.

Based on the results obtained, enzymatic hydrolysis under stirring (dynamic conditions) was selected as the optimal setup for producing BCNPs, as it yielded a more homogeneous sample. To further refine the recovery of BCNPs under these conditions, centrifugation was performed at increasing g‐forces, that is, 4,000–8,000–10,000–14,000–27,000 g, while all other parameters remained consistent with the previously outlined experimental procedure.

After centrifugation, all the samples were analyzed by DLS. The sizes of the BCNPs (ranging from 250 to 400 nm) were consistent across all centrifugation speeds after 24 h of enzymatic hydrolysis, suggesting that the centrifugation speed had small impact on the size of the BCNPs (Figure 2D and Table 3). However, at the lowest g‐force (4,000 g), a higher variability in nanoparticle size was observed, with BCNPs measuring 331 ± 102 nm and a polydispersity index (PDI) of 0.936 ± 0.093, indicating high variability. In contrast, BCNPs collected at 27,000 *g* exhibited the smallest size (253 ± 60 nm) and the lowest PDI (0.789 ± 0.233), suggesting a more homogeneous sample with less tendency to aggregate.

**TABLE 3:** DLS analyses of BCNPs collected at increasing g‐forces upon enzymatic hydrolysis in dynamic conditions.

g‐Force	Major peak intensity (%)	Major peak size (nm)	Z‐average (nm)	PDI
4000	100	331 ± 102	1779 ± 388	0.936 ± 0.093
8000	100	405 ± 59	1244 ± 225	0.826 ± 0.126
10,000	97 ± 6	347 ± 55	1034 ± 333	0.782 ± 0.135
14,000	100	427 ± 54	1737 ± 457	0.724 ± 0.373
27,000	87 ± 20	253 ± 60	1166 ± 736	0.789 ± 0.233

A decrease of PDI value, indicative a higher homogeneity of the sample, was also observed at 10,000 g (PDI of 0.782 ± 0.135) and 14,000 g (PDI of 0.724 ± 0.373). In both cases, the BCNPs sizes were similar, that is, 347 ± 55 and 427 ± 54 nm for 10,000 and 14,000 *g*, respectively. Based on these findings, the BCNPs recovered at 10,000 *g* were selected to perform further analyses.

#### 3.1.3 Production of BCNPs in Dynamic Conditions by Using Higher Substrate Concentrations

To further optimize BCNPs production, increasing concentrations of BC macrofibers were used as substrates for the hydrolysis, performed with 20 U/mL of cellulases from *Trichoderma reesei*. Specifically, 1–2–4 mg/mL of BC macrofibers were subjected to enzymatic hydrolysis under dynamic conditions and recovered by centrifugation at 10,000 and 14,000 *g*. The size of the major peak for samples with 2 mg/mL substrate (481 ± 71 nm after centrifugation at 10,000 *g*; 418 ± 126 nm after centrifugation at 14,000 *g*) was slightly higher than that for 1 mg/mL substrate (363 ± 65 nm at 10,000 *g*; 427 ± 54 nm at 14,000 *g*), as shown in Table S3. No significant changes were observed in Z‐average values, although the PDI improved, particularly for BCNPs recovered at 10,000 *g* (PDI = 0.433 ± 0.237).

To increase the BCNPs yield, 4 mg/mL of BC macrofibers were also used as substrate. In this case, although the size of the major peaks of the samples recovered at 10,000 *g* (416 ± 179) and 14,000 *g* (458 ± 97 nm) was similar to those obtained in the previous conditions tested, a significant increase in PDI, especially for the sample collected at 10,000 *g* (PDI = 0.934 ± 0.067), indicated higher sample heterogeneity under these experimental conditions (Table S3). This was also further confirmed by the appearance of the supernatant after centrifugation at 14,000 *g* (Figure S3A,B), which clearly showed the presence of undigested BC fibers. Based on these results, further experiments were conducted using BCNPs derived from the hydrolysis of 1 mg/mL BC macrofibers under dynamic conditions for 24 h at 40°C, followed by BCNPs recovery through centrifugation at 10,000 *g* for 30 min. Notably, the obtained results and BCNPs size align with recent findings on the production of BC nanocrystals via enzymatic hydrolysis [70, 71, 72].

### 3.2 Evaluation of Storage Stability of Produced BCNPs

To assess the stability of the produced BCNPs, analyses were performed on samples stored at room temperature for increasing time intervals (7–14–20–30 days). After each storage period, samples were analyzed by DLS. As shown in Table 4, for the sample stored for 7 days, the size of the major peak remained identical to that observed at time 0 (BCNPs recovered immediately after hydrolysis under dynamic conditions), indicating that no aggregation occurred during the 7‐day incubation at room temperature.

**TABLE 4:** DLS analyses of BCNPs stored for increasing time intervals.

Incubation time (days)	Major peak intensity (%)	Major peak size (nm)	Z‐average (nm)	PDI
0	97 ± 6	347 ± 55	1035 ± 333	0.782 ± 0.135
7	98 ± 5	349 ± 67	1637 ± 527	0.852 ± 0.229
14	100 ± 0.1	460 ± 93	1694 ± 535	0.715 ± 0.257
20	100 ± 0.1	519 ± 83	1605 ± 877	0.524 ± 0.360
30	100 ± 0.1	469 ± 255	2309 ± 1004	0.807 ± 0.306

However, variations were observed when BCNPs were stored for 14 days (Table 4). Specifically, the major peak size increased from 349 ± 67 nm (after 7 days) to 460 ± 93 nm (after 14 days). A further increase in the major peak size was noted after 20 days of storage, reaching 519 ± 83 nm, likely due to a progressive aggregation of BCNPs over time.

After 30 days of storage, the presence of large aggregates was suggested by the increase in the Z‐average value from 1605 ± 877 nm (after 20 days) to 2309 ± 1004 nm (after 30 days), although no further increase in the major peak size was observed compared to the 20‐day storage period (Table 4).

#### 3.2.1 Analysis of the Effects of the Sonication on Stored BCNPs

To verify the possibility of dissolving the aggregates formed during the storage, the same kind of analyses was performed after sonicating the BCNPs. It was demonstrated that a mild sonication step reduced the major peak size of BCNPs stored for 20 days, from 519 ± 83 nm (without sonication, Table 4) to 434 ± 83 nm (with sonication, Table 5), indicating that sonication can help to dissolve the aggregates formed after 20 days of incubation at room temperature.

**TABLE 5:** DLS analyses of BCNPs stored for increasing time intervals and sonicated.

Time (days)	Major peak intensity (%)	Major peak size (nm)	Z‐average (nm)	PDI
0	97 ± 6	347 ± 55	1035 ± 333	0.782 ± 0.135
7	100	364 ± 93	1170 ± 243	0.802 ± 0.107
14	99 ± 2	399 ± 79	1475 ± 609	0.703 ± 0.297
20	100	434 ± 83	1753 ± 587	0.483 ± 0.374
30	98 ± 4	560 ± 54	1655 ± 550	0.857 ± 0.142

However, it is important to note that a mild sonication step was not effective in dissolving the aggregates formed after 30 days of storage. In this case, the major peak size was 560 ± 54 nm with sonication (Table 5) and 469 ± 255 nm without sonication (Table 4). This suggests that a stronger sonication might be required to dissolve the aggregates formed after 30 days of storage.

**FIGURE 3:**
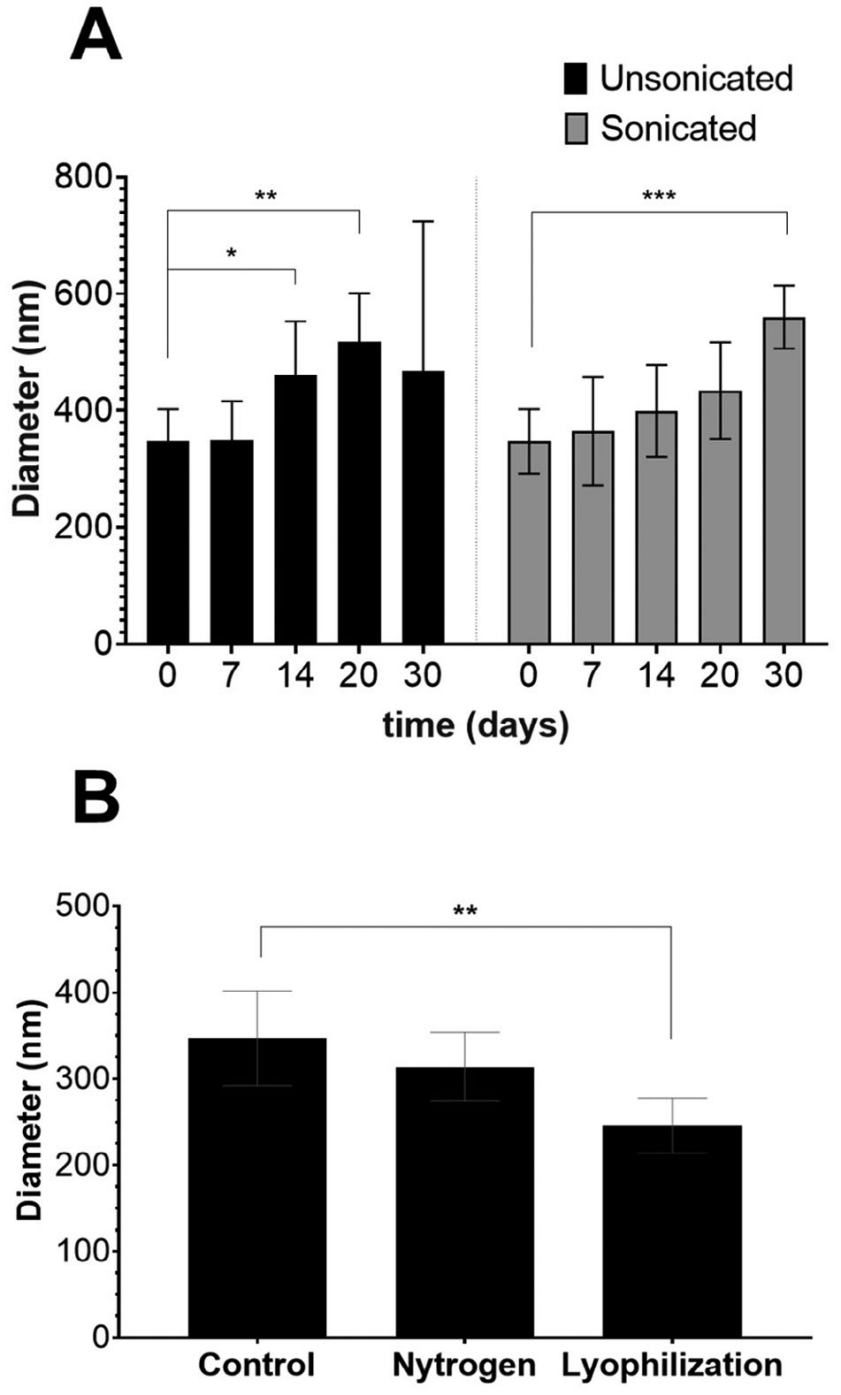
Storage stability of obtained BCNPs. (A) Diameter of BCNPs stored for increasing time intervals without (unsonicated) and with (sonicated) repeated rounds of sonication in an ultrasound bath. (B) Diameter of BCNPs desiccated under nitrogen gas flow or by lyophilization. Significant differences were indicated as *p < 0.05, **p < 0.01, or ***p < 0.001.

Based on these results, it can be concluded that BCNPs can be stored at room temperature for up to 7 days without changes in their size compared to the sample collected at time 0 (Figure 3A). Additionally, if a mild sonication step is applied, the storage period can be extended to 20 days without altering the properties of the BCNPs (Tables 4 and 5).

#### 3.2.2 Evaluation of the Effectiveness of Alternative Strategies to Store Produced BCNPs

To develop an efficient strategy for storing BCNPs, alternative procedures were also explored. Specifically, BCNPs recovered by centrifugation after hydrolysis were desiccated either under nitrogen flow (condition A) or by lyophilization (condition B). After desiccation, the dried BCNPs were resuspended, sonicated, and analyzed by DLS. The results indicated that both strategies could effectively preserve BCNPs, as no significant variations in the major peak size were observed following desiccation, either under nitrogen flow or by lyophilization (values ranged from 246 ± 32 to 347 ± 55 nm, as shown in Table S4).

For BCNPs desiccated under nitrogen flow, a slight decrease in the major peak size (314 ± 40 nm) and a corresponding decrease in the major peak intensity value (87 ± 27 intensity percentage) were observed (sample A in Table S4 and Figure 3B). This suggests a probable loss of BCNPs due to their attachment to the plastic surface during desiccation. A similar phenomenon was noted for BCNPs desiccated by lyophilization (sample B in Table S4 and Figure 3B). In this case, a decrease in size by approximately 100 nm was observed (246 ± 32 nm in Table S4), with the major peak intensity remaining close to 100% (Table S4).

Based on these results, it can be concluded that desiccation, if optimized, may represent a suitable method for storing BCNPs. Currently, BNCPs are mostly produced from plant‐derived biomasses, which require harsh mechanical and chemical pre‐treatment steps to improve cellulose purity and reduce fiber length. In contrast to plant‐derived cellulose, BC fibrils are produced through a bottom‐to‐top approach, resulting in a robust network of naturally occurring cellulose nanofibers with an average diameter of 10–100 nm [74]. This unique characteristic, combined with the physical properties of BC, provides enhanced accessibility to glycolytic enzymes, which is crucial for applications requiring cellulose nanoparticles with both nanosized diameter and length. While cellulose is typically subjected to physical defibrillation and acid hydrolysis using highly concentrated sulfuric or hydrochloric acid at high temperatures, [75] increasing studies between 2013 and 2021 have proposed the use of commercially available enzymes as an environmentally friendly approach to reduce fiber length [76]. Among the proposed enzymes, endo‐1,4‐β‐glucanases from *Thermobifida halotolerans* and cellulases from *Trichoderma reesei* have been suggested to produce nanoparticles with a length of 1.8–2.0 µm and a width of 250–350 nm [77]. The disadvantages of enzymatic hydrolysis are associated with the formation of a more heterogeneous system and the potential impact of longer incubation times on the yield, due to the further hydrolysis of cellulose into cellobiose and glucose units [78]. It should be noted that, despite significant efforts by the scientific community, a standardized method for producing bacterial nanocellulose via enzymatic hydrolysis has yet to be established. The development of sustainable enzymatic digestion of BC is hindered by the lack of commercially available enzyme formulations specifically designed for this process [70, 72, 79]. Here, a clear and reliable method for the green biotechnological production, harvesting, and storage of bacterial cellulose nanoparticles (BCNPs) in a short timeframe is outlined.

### 3.3 Functionalization of Produced BCNPs With a Human Antimicrobial Peptide

The possibility of functionalizing produced BCNPs with human antimicrobial peptides has also been exploited. To this end, a human antimicrobial peptide previously characterized by our research group and found to possess promising antimicrobial properties [56, 57], here named r(P)ApoB_L_^Pro^, was selected as a model peptide.

To establish the loading procedure, the amount of BCNPs obtained under dynamic conditions and collected by centrifugation was first evaluated using QCM analyses. BCNPs obtained by enzymatic hydrolysis under dynamic conditions were centrifuged at increasing *g*‐forces (i.e., 10,000, 14,000, and 27,000 *g*) as previously described, and the number of recovered BCNPs was estimated by QCM analysis.

**FIGURE 4:**
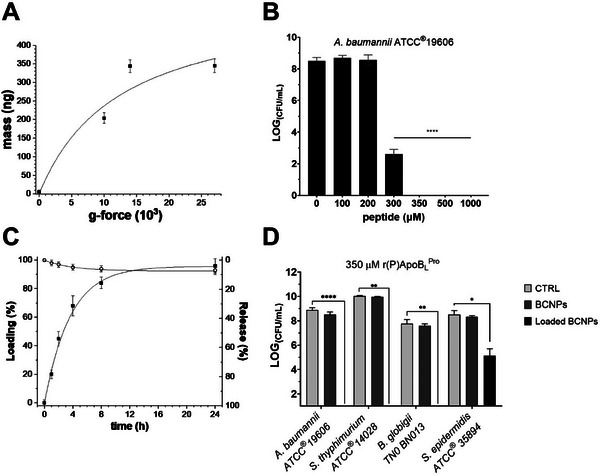
Antimicrobial properties of obtained BCNPs loaded with r(P)ApoB_L_^Pro^ human antimicrobial peptide. (A) QCM analyses to determine the amount of recovered BCNPs upon centrifugation at 10,000, 14,000, and 27,000 *g*. (B) Antimicrobial activity of recovered 5× concentrated BCNPs loaded with increasing concentrations (100–200–300–350–500–1,000 µM) of r(P)ApoB_L_^Pro^ peptide on *A. baumannii* ATCC19606. (C) Loading and release of peptide molecules over time determined by QCM analyses. (D) Antimicrobial activity of recovered 5× concentrated BCNPs loaded with 350 µM r(P)ApoB_L_^Pro^ peptide on *A. baumannii* ATCC19606, *S. typhimurium* ATCC14028, *B. globigii* TNO BM013, and *S. epidermidis* ATCC35894. Significant differences were indicated as *p < 0.05, **p < 0.01, or ****p < 0.0001.

As shown in Figure 4A, approximately 200 and 350 ng of BCNPs were recovered upon centrifugation at 10,000 and 14,000 *g*, respectively. Increasing the *g*‐force to 27,000 *g* did not result in any further increase in the amount of recovered BCNPs compared to 14,000 *g* (Figure 4A). Based on these results, BCNPs to be loaded with r(P)ApoB_L_^Pro^ antimicrobial peptide were obtained by enzymatic hydrolysis under dynamic conditions, followed by centrifugation at 14,000 *g* for 30 min at 25°C.

The resulting sediment (loaded BCNPs) was then resuspended in a volume of Milli‐Q water five times smaller than the initial volume (5× BCNPs) and incubated with increasing concentrations of r(P)ApoB_L_^Pro^ antimicrobial peptide (0–100–200–300–350–500–1,000 µM) for 24 h under stirring. After incubation, the sample was centrifuged to separate the functionalized BCNPs (sediment) from the unbound peptide molecules (supernatant).

#### 3.3.1 Evaluation of the Antimicrobial Properties of BCNPs Loaded With a Human Antimicrobial Peptide on *A. baumannii* ATCC19606 Bacterial Strain

Obtained samples were then tested for their antimicrobial properties against *A. baumannii* ATCC19606. This bacterial strain was selected due to its high susceptibility to the antimicrobial activity of the peptide (MBC = 5 µM). The results revealed that BCNPs loaded with 350, 500, and 1,000 µM r(P)ApoB_L_^Pro^ antimicrobial peptide completely inhibited bacterial growth, while BCNPs loaded with 300 µM r(P)ApoB_L_^Pro^ antimicrobial peptide caused a 70% inhibition of bacterial growth (Figure 4B). No effect on bacterial cell viability was observed when BCNPs loaded with 100 and 200 µM r(P)ApoB_L_^Pro^ antimicrobial peptide were tested (Figure 4B).

It should be emphasized that the peptide molecules immobilized on the nanoparticle surface retain their bactericidal effect, although the random adsorption of peptide molecules onto the nanoparticle surface, combined with spatial constraints, necessitates higher peptide concentrations to ensure effective bacterial cell killing, compared to the use of the peptide in solution.

#### 3.3.2 Analysis of the Ability of BCNPs to Load and Release the Selected Human Antimicrobial Peptide

Since 350 µM r(P)ApoB_L_^Pro^ antimicrobial peptide was identified as the lowest peptide concentration capable of completely inhibiting bacterial growth, BCNPs loaded with 350 µM r(P)ApoB_L_^Pro^ antimicrobial peptide were selected for further analysis.

First, kinetic analyses were performed to evaluate the efficiency of the produced BCNPs in loading r(P)ApoB_L_^Pro^ antimicrobial peptide. To this end, 5× BCNPs were incubated with 350 µM r(P)ApoB_L_^Pro^ antimicrobial peptide for 24 h under stirring. At defined time intervals (0–1–2–4–8–24 h), aliquots of the sample were withdrawn, centrifuged at 14,000 *g* for 30 min at 25°C, and the collected supernatants, containing unbound peptide molecules, were analyzed by QCM to determine the amount of peptide loaded onto BCNPs at increasing time intervals.

The results showed that 84% ± 4% of the peptide molecules were absorbed onto the surface of BCNPs during 8 h of incubation, reaching a maximum of 96% ± 5% loading after 24 h (Figure 4C).

Considering that, under the experimental conditions tested, BCNPs likely load r(P)ApoB_L_^Pro^ antimicrobial peptide through electrostatic interactions between the positively charged peptide molecules and the negatively charged BCNPs surface, the release of the peptide from loaded BCNPs was also evaluated by QCM analyses over time. To this end, after incubating 5× BCNPs with 350 µM r(P)ApoB_L_^Pro^ antimicrobial peptide for 24 h under stirring, the sample was centrifuged at 14,000 *g* for 30 min at 25°C. The sediment containing the functionalized BCNPs was then resuspended in the initial volume of Milli‐Q water and incubated for 24 h under stirring.

At defined time intervals (0–1–2–4–8–24 h), aliquots of the sample were withdrawn, centrifuged at 14,000 *g* for 30 min at 25°C, and the supernatants, which likely contained released peptide molecules, were analyze by QCM to determine the percentage of released peptide. As shown in Figure 4C, a small portion of the peptide molecules (about 7% ± 3%) was released from BCNPs after 24 h of incubation.

The proposed system was developed without the need for additional chemical modifications of either the peptide or the nanoparticles, resulting in a straightforward strategy applicable to a broad range of natural and unmodified antimicrobial peptides. Unlike previously reported strategies that rely on chemical modifications of cellulose or custom peptide synthesis [80, 81], this approach offers a significant advantage by preserving the native structure and bioactivity of the peptide molecules, while simultaneously enhancing their stability and efficiency through the nanoparticle‐based system. The absence of chemical modifications also simplifies the preparation process, making this strategy potentially scalable and suitable for diverse applications involving unmodified peptides.

Furthermore, unlike several other PLA‐ and chitosan‐based nanoparticles, which are associated with burst release of peptide molecules within hours after loading [82], the BCNPs developed in this study were able to fully load the r(P)ApoB_L_^Pro^ peptide, showing no significant release within 24 h of incubation at room temperature in water. Regarding this aspect, it should be emphasized that, to monitor peptide loading, the obtained BCNPs were incubated with the selected human antimicrobial peptide molecules for defined time intervals, and the samples were centrifuged at the end of each incubation. QCM analyses were then performed to quantify the amount of peptide in the supernatant at selected time intervals during the loading process. Since no detectable free peptide molecules remained in the supernatant after 24 h of incubation, it was concluded that the antimicrobial peptide molecules were fully loaded onto the BCNPs.

Furthermore, to assess the stability of the peptide entrapment, the collected functionalized nanoparticles were incubated in water, and peptide release was monitored over time. Considering that the samples were incubated at room temperature under continuous shaking, any instability in peptide loading would likely have resulted in a significant release of peptide molecules in the initial hours. Instead, QCM data indicated no significant release of peptide molecules within the first 24 h, supporting the conclusion that the peptide molecules were stably retained on the surface of the nanoparticles under the tested experimental conditions.

This stable adsorption of peptide molecules onto BCNPs is likely mediated by weak, stable interactions, probably electrostatic interactions between the positively charged antimicrobial peptide molecules and the negatively charged surface of BCNPs. Although these weak interactions ensure the stable association of peptide molecules with the nanoparticles under the experimental conditions tested, it cannot be excluded that an effective release of peptide molecules could occur under conditions resembling the physiological environment, such as different pH, ionic strength, or temperature values.

#### 3.3.3 Evaluation of the Antimicrobial Properties of BCNPs Loaded With a Human Antimicrobial Peptide on *S. typhimurium* ATCC14028, *B. globigii* TNO BM013, and *S. epidermidis* ATCC35894 Bacterial Strains

To further investigate the antimicrobial properties of BCNPs loaded with 350 µM r(P)ApoB_L_^Pro^ antimicrobial peptide, analyses were also performed on *S. typhimurium* ATCC14028, *B. globigii* TNO BM013, and *S. epidermidis* ATCC35894. Similar to the observations collected in the case of *A. baumanii* ATCC19606 (Figure 4B), BCNPs loaded with 350 µM r(P)ApoB_L_^Pro^ antimicrobial peptide were found to completely inhibit the growth of *S. typhimurium* ATCC14028 and *B. globigii* TNO BM013 (Figure 4D). However, in the case of *S. epidermidis* ATCC35894, approximately 40% inhibition of bacterial growth was detected (Figure 4D).

#### 3.3.4 Characterization of BCNPs Loaded With Selected Human Antimicrobial Peptide

**FIGURE 5:**
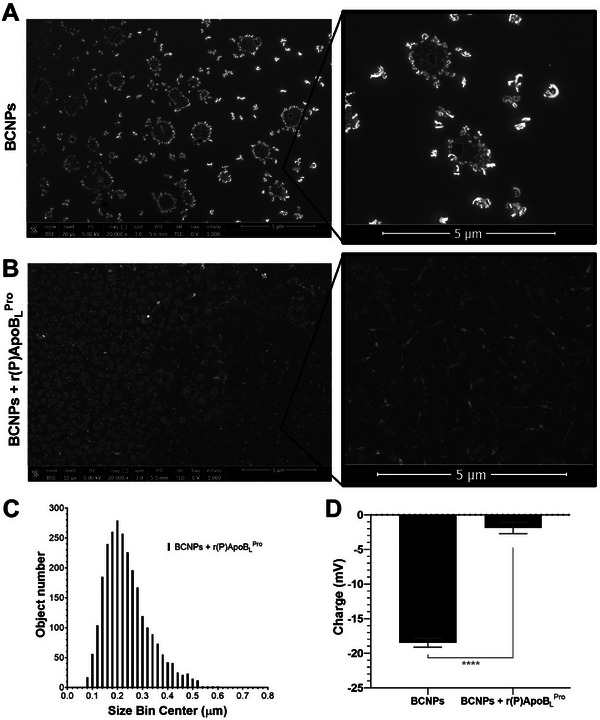
SEM analyses of unfunctionalized BCNPs and of BCNPs functionalized with 350 µM r(P)ApoB_L_^Pro^ human antimicrobial peptide. (A) Unfunctionalized BCNPs. (B) BCNPs functionalized with 350 µM r(P)ApoB_L_^Pro^ human antimicrobial peptide. (C) Data obtained by counting the number of objects with a specific size in different acquisition fields. (D) Charge of BCNPs before and after peptide loading. Significant differences were indicated as *p < 0.05, **p < 0.01, or ****p < 0.0001.

To characterize BCNPs loaded with 350 µM r(P)ApoB_L_^Pro^ antimicrobial peptide, SEM analyses were also performed (Figure 5). A strong tendency for aggregation was observed in the case of unfunctionalized BCNPs (Figure 5A), consistent with previously reported DLS and SEM analyses (Figure 2A,B). The tendency to aggregate, however, was abolished when BCNPs were functionalized with 350 µM r(P)ApoB_L_^Pro^ antimicrobial peptide (Figure 5B), likely due to the formation of electrostatic interactions between negatively charged surface of the BCNPs and positively charged peptide molecules.

To further confirm the effect of the peptide, the number of BCNPs with a specific size was also counted by analyzing different acquisition fields. The data were plotted by reporting the number of counted objects as a function of their size (Figure 5C). It was found that most of the BCNPs had a size of about 200 nm when loaded with r(P)ApoBL^Pro^ peptide, confirming that the presence of the peptide prevents the aggregation of BCNPs observed in the absence of the peptide (Figure 5C).

Furthermore, the charge of the loaded BCNPs was determined by Zeta potential analyses. A shift in Zeta potential values from –18.5 ± 0.6 mV to –1.9 ±0.8 mV was detected upon loading with peptide molecules (Figure 5D), likely due to the formation of electrostatic interactions between the negatively charged surface of the BCNPs and the positively charged peptide molecules.

These results highlight the possibility of functionalizing BCNPs with antimicrobial peptide molecules through non‐covalent binding. This finding aligns with recent studies suggesting that the absorption of the nisin peptide on BC membranes via non‐covalent binding enhances the antimicrobial properties of nisin [73].

### 3.4 Biocompatibility of BCNPs Functionalized With r(P)ApoB_L_^Pro^ Human Antimicrobial Peptide

**FIGURE 6:**
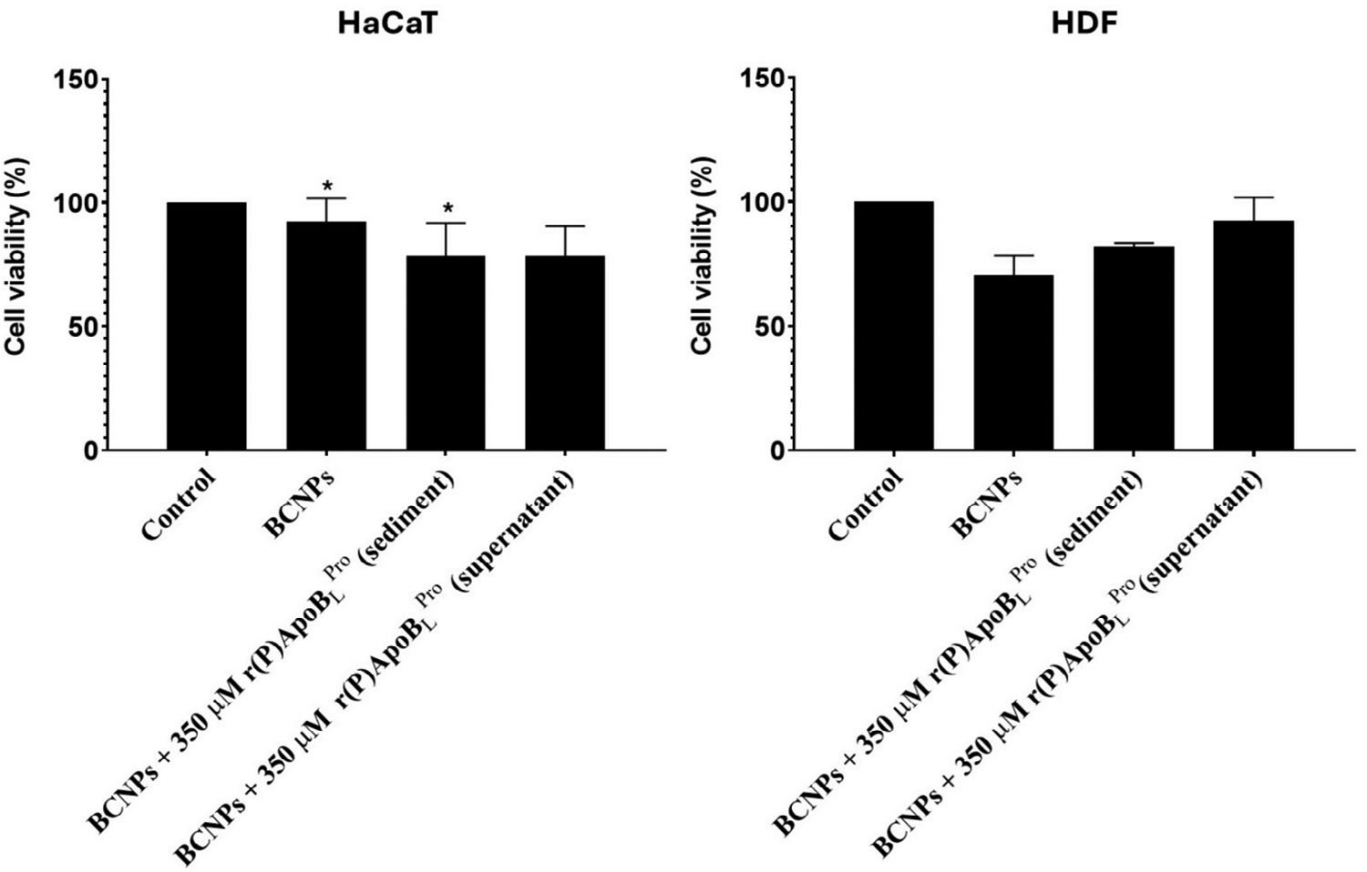
Biocompatibility of unfunctionalized BCNPs and of BCNPs loaded with 350 µM r(P)ApoB_L_^Pro^ antimicrobial peptide. Analysis of the effects of unfunctionalized BCNPs, of BCNPs loaded with 350 µM r(P)ApoB_L_^Pro^ antimicrobial peptide (sediment), and of unbound peptide molecules (supernatant) on the viability of HaCaT immortalized human keratinocytes (A) and of HDF human dermal fibroblasts (B). Cell viability was assessed by MTT assays and expressed as the percentage of viable cells with respect to non‐treated cells. Experiments were performed three times. Error bars represent the standard deviation of the mean. Significant differences were indicated as *p < 0.05 for treated versus control samples.

To assess the safety of BCNPs functionalized with r(P)ApoB_L_^Pro^ human antimicrobial peptide toward host cells, their effects on the viability of cultured human cells were tested. To do this, primary human dermal fibroblasts (HDFs) and HaCaT immortalized human keratinocytes were incubated for 24 h at 37°C either in the presence of the sediment obtained by centrifugating 5× concentrated BCNPs incubated with 350 µM r(P)ApoB_L_^Pro^ peptide, or in the presence of the supernatant (Figure 6). It should be noted that the sediment is expected to contain only functionalized BCNPs, while the supernatant likely contains only unbound peptide molecules.

Cell viability was assessed by the MTT reduction assay, as an indicator of metabolically active cells. Viability values are expressed as the percentage of viable cells in the treated samples relative to control cells grown in the absence of tested molecules. As shown in Figure 6, most of the cells treated with BCNPs, either present in the sediment containing the majority of the functionalized BCNPs or in the supernatant containing the unbound peptide molecules and a small portion of uncollected BCNPs, were found to be metabolically active. The percentage of viable cells was approximately 80% for HaCaT cells (Figure 6A) and 80% for HDF cells (Figure 6B), respectively.

## 4 Conclusions

BCNPs were selected as a suitable system for loading AMPs due to their excellent biocompatibility and hygroscopicity, key features that make them promising biomaterials for biomedical and healthcare applications. *Komagataeibacter xylinus*, grown in Hestrin–Schramm medium, was chosen as the suitable host for producing BC. The obtained BC macrofibers were subjected to enzymatic hydrolysis to generate BCNPs through a sustainable, green biotechnological process.

Once obtained, the BCNPs were evaluated for their amount, size, charge, morphology, and stability. The BCNPs were also loaded with a human antimicrobial peptide via non‐covalent binding. This peptide, previously identified in human apolipoprotein B, was recombinantly produced and shown to possess strong antimicrobial properties.

The functionalized BCNPs were found to completely inhibit the growth of the bacterial strains under study and demonstrated good biocompatibility profiles. In conclusion, eco‐friendly, biodegradable antimicrobial BCNPs, loaded via non‐covalent binding with a human antimicrobial peptide, have been successfully produced. The results open interesting perspectives for using the produced BCNPs as a suitable system for developing effective antimicrobial formulations.

## Author Contributions

**Martina Schibeci**: conceptualization (equal), data curation (lead), formal analysis (equal), investigation (lead), methodology (lead), writing–original draft (equal), writing–review and editing (equal). **Rosa Gaglione**: conceptualization (equal), data curation (equal), formal analysis (equal), investigation (equal), methodology (equal), writing–original draft (equal), writing–review and editing (equal). **Noemi Russo**: Conceptualization (equal), data curation (equal), formal analysis (equal), investigation (equal), methodology (equal), writing–original draft (equal), writing–review and editing (equal). **Raffaele Velotta**: conceptualization (equal), data curation (equal), formal analysis (equal), investigation (equal), methodology (equal), writing–original draft (equal), writing–review and editing (equal). **Bartolomeo Della Ventura**: conceptualization (equal), funding acquisition (equal), project administration (equal), supervision (equal), writing–review and editing (equal). **Angela Arciello**: conceptualization (lead), funding acquisition (lead), project administration (lead), supervision (lead), writing–review and editing (lead). The manuscript was written through the contributions of all authors. All authors have given approval to the final version of the manuscript.

## Conflicts of Interest

The authors declare no conflicts of interest.

## Supporting information



Supporting Information

## Data Availability

The data that supports the findings of this study are available in the supplementary material of this article.
