# Supplementary material for: Enzymatically Crafted Bacterial Cellulose Nanoparticles Functionalized With Antimicrobial Peptides: Toward Sustainable Antimicrobial Formulations
Source: Biotechnol J. 2025 Feb 24;20(2):e202400573. doi: 10.1002/biot.202400573 (PMC11848709; doi:10.1002/biot.202400573)
Supplement: Supplementary file 1 — Supporting Information [file BIOT-20-e202400573-s001.docx]

**Title**: Enzymatically crafted Bacterial Cellulose Nanoparticles functionalized with Antimicrobial Peptides: towards sustainable antimicrobial formulations

Martina Schibeci^1^, Rosa Gaglione^1,2^, Noemi Russo^1^, Raffaele Velotta^3^, Bartolomeo Della Ventura^3^, Angela Arciello^1,2,^*

^1^Department of Chemical Sciences, University of Naples Federico II, Via Cintia 21, I-80126 Naples

^2^Istituto Nazionale di Biostrutture e Biosistemi (INBB), Rome, Italy

^3^Department of Physics “Ettore Pancini”, University of Naples Federico II, Via Cintia 21, I-80126, Naples, Italy

**Correspondence** Angela Arciello, Department of Chemical Sciences, University of Naples Federico II, Via Cintia 21, I-80126, Naples, Italy; Istituto Nazionale di Biostrutture e Biosistemi (INBB), Rome, Italy. Email: anarciel@unina.it

KEYWORDS: bacterial cellulose nanoparticles, *Komagataeibacter xylinus*, sustainable production processes, cellulose enzymatic hydrolysis, host defence peptides, antimicrobial formulations.


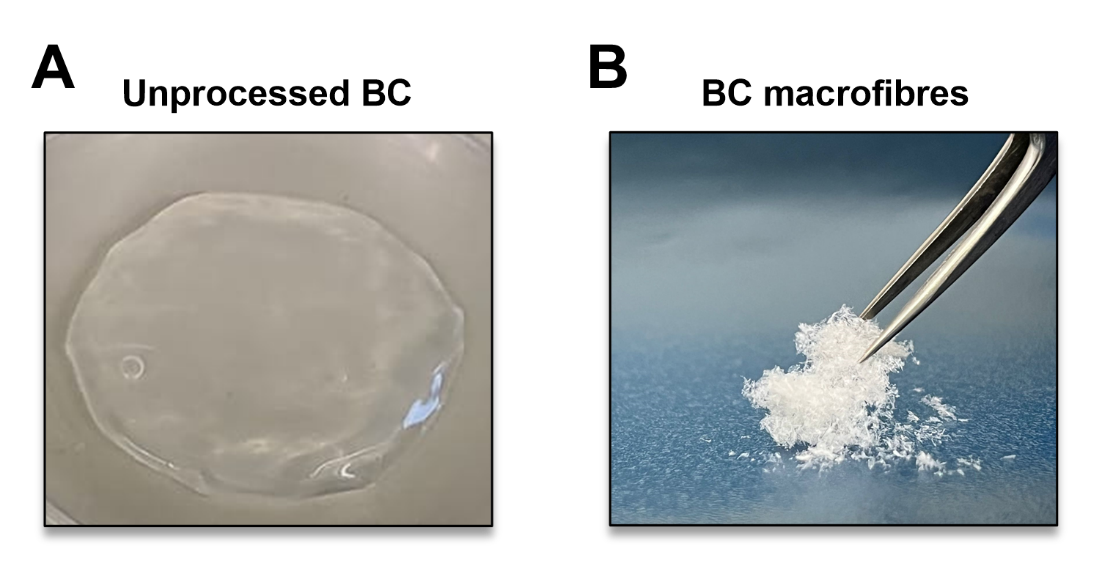


**Supplementary Figure S1**. (A) Image of BC pellicle produced by bacterial cells. (B) BC macrofibres obtained upon pulping, freeze-drying and grinding of the wet BC pellicle.

| **Supplementary Table S1. Analysis of the samples obtained upon hydrolysis in static conditions and centrifugation.** | | | | | | | | | | |  |
| --- | --- | --- | --- | --- | --- | --- | --- | --- | --- | --- | --- |
| **Fraction** | | **Major  Peak Intensity  (%)** | **Major  Peak Size  (nm)** | | **Secondary Peak Intensity  (%)** | **Secondary Peak Size  (nm)** | | **Z-Average  (nm)** | **PDI** | |  |
| Reaction mixture | | 100 | 2,463 ± 237 | | / | / | | 3,223 ± 259 | 0.363 ± 0.019 | |  |
| Reaction supernatant | | 74 ± 1 | 8 ± 1 | | 26 ± 1 | 580 ± 278 | | 231 ± 202 | 0.371 ± 0.099 | |  |
| Wash supernatant | | 87 ± 6 | 410 ± 327 | | 12 ± 6 | 5 ± 1 | | 1,073 ± 529 | 0.646 ± 0.01 | |  |
| Sediment | | 100 | 767 ± 27 | | / | / | | 2,856 ± 703 | 0.724 ± 0.054 | |  |
|  | | | | | | | | | | |  |
| **Supplementary Table S2. Analyses of the sediments obtained by centrifugation upon 3 and 24 h of hydrolysis in dynamic conditions.** | | | | | | | | | | | |
| **time (hours)** | **Major peak intensity (%)** | | | **Major peak size (nm)** | | | **Z-average (nm)** | | | **PDI** | |
| **3** | 100 | | | 244 ± 95 | | | 2,282 ± 1,683 | | | 0.856 ± 0.229 | |
| **24** | 100 | | | 391 ± 67 | | | 1,607 ± 301 | | | 0.813 ± 0.148 | |
|  | | | | | | | | | | | |


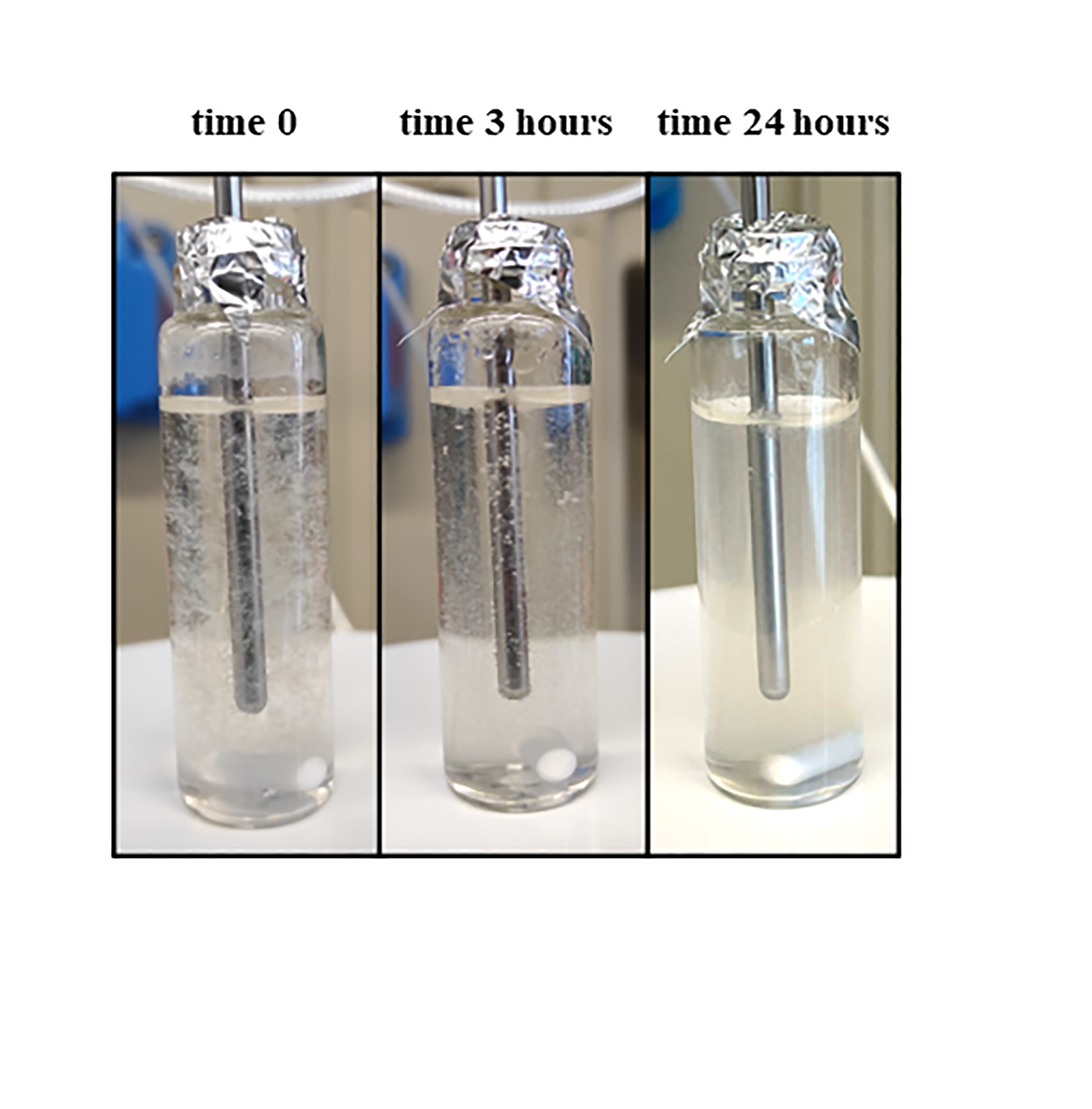


**Supplementary Figure S2**. Photos of hydrolysis reaction performed under stirring (time 0, after 3 h and after 24 h) were acquired over time.

| **Supplementary Table S3. DLS analyses of BCNPs obtained by using increasing concentrations of BC macro-fibers.** | | | | | |
| --- | --- | --- | --- | --- | --- |
| **Concentration (mg/mL)** | ***g-force*** | **Major peak intensity (%)** | **Major peak size (nm)** | **Z-average (nm)** | **PDI** |
| 1 | 10,000 | 97 ± 6 | 347 ± 55 | 1,034 ± 333 | 0.782 ± 0.135 |
| 1 | 14,000 | 100 | 427 ± 54 | 1,737 ± 457 | 0.724 ± 0.373 |
| 2 | 10,000 | 100 | 481 ± 71 | 917 ± 65 | 0.433 ± 0.237 |
| 2 | 14,000 | 100 | 418 ± 126 | 1,278 ± 347 | 0.696 ± 0.270 |
| 4 | 10,000 | 100 | 416 ± 179 | 2,279 ± 622 | 0.934 ± 0.067 |
| 4 | 14,000 | 100 | 458 ± 97 | 1,626 ± 662 | 0.761 ± 0.263 |
| **SD is not shown when lower than 0.01%.** | | | | | |


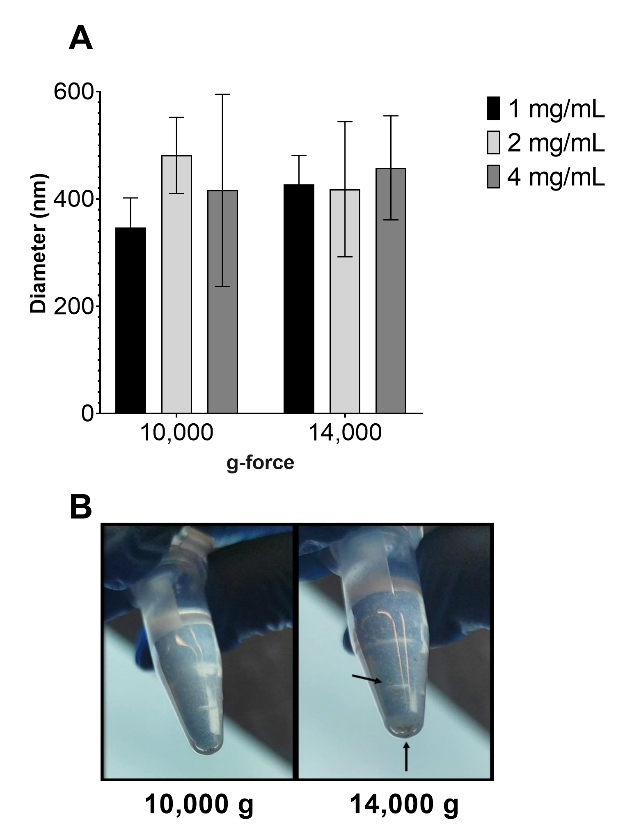


**Supplementary Figure S3**. (A) Diameter of BCNPs obtained by hydrolyzing increasing concentrations of BC macro-fibers and recovered by centrifugation at 10,000 or 14,000 g. (B) Photos of sediments and supernatants obtained upon enzymatic hydrolysis of BC macro fibers (4 mg/mL) and subsequent centrifugation at 10,000 or 14,000 g.

| **Supplementary Table S4. Analyses of BCNPs desiccated under nitrogen or by lyophilization.** | | | | |
| --- | --- | --- | --- | --- |
| **Sample** | **Major peak intensity (%)** | **Major peak Size (nm)** | **Z-average (nm)** | **PDI** |
| Control | 97 ± 6 | 347 ± 55 | 1,035 ± 333 | 0.782 ± 0.135 |
| Sample A | 87 ± 27 | 314 ± 40 | 1,157 ± 480 | 0.831 ± 0.126 |
| Sample B | 100 ± 2 | 246 ± 32 | 1,099 ± 570 | 0.712 ± 0.146 |
|  | | | | |
